# 2012-2013 Seasonal Influenza Vaccine Effectiveness against Influenza Hospitalizations: Results from the Global Influenza Hospital Surveillance Network

**DOI:** 10.1371/journal.pone.0100497

**Published:** 2014-06-19

**Authors:** Joan Puig-Barberà, Angels Natividad-Sancho, Odile Launay, Elena Burtseva, Meral A. Ciblak, Anita Tormos, Amparo Buigues-Vila, Sergio Martínez-Úbeda, Anna Sominina, the GIHSN Group

**Affiliations:** 1 Foundation for the Promotion of Health and Biomedical Research in the Valencia Region FISABIO – Public Health, Valencia, Spain; 2 Université Paris Descartes, Sorbonne Paris Cité, Inserm, CIC 1417 and the French Vaccine Research Network (REIVAC), Paris, France; 3 D.I. Ivanovsky Institute of Virology, Moscow, Russian Federation; 4 National Influenza Reference Laboratory Cappa-Istanbul, Istanbul, Turkey; 5 Research Institute of Influenza, St. Petersburg, Russian Federation; Centers for Disease Control and Prevention, United States of America

## Abstract

**Background:**

The effectiveness of currently licensed vaccines against influenza has not been clearly established, especially among individuals at increased risk for complications from influenza. We used a test-negative approach to estimate influenza vaccine effectiveness (IVE) against hospitalization with laboratory-confirmed influenza based on data collected from the Global Influenza Hospital Surveillance Network (GIHSN).

**Methods and Findings:**

This was a multi-center, prospective, active surveillance, hospital-based epidemiological study during the 2012–2013 influenza season. Data were collected from hospitals participating in the GIHSN, including five in Spain, five in France, and four in the Russian Federation. Influenza was confirmed by reverse transcription-polymerase chain reaction. IVE against hospitalization for laboratory-confirmed influenza was estimated for adult patients targeted for vaccination and who were swabbed within 7 days of symptom onset. The overall adjusted IVE was 33% (95% confidence interval [CI], 11% to 49%). Point estimates of IVE were 23% (95% CI, −26% to 53%) for influenza A(H1N1)pdm09, 30% (95% CI, −37% to 64%) for influenza A(H3N2), and 43% (95% CI, 17% to 60%) for influenza B/Yamagata. IVE estimates were similar in subjects <65 and ≥65 years of age (35% [95% CI, −15% to 63%] vs.31% [95% CI, 4% to 51%]). Heterogeneity in site-specific IVE estimates was high (I^2^ = 63.4%) for A(H1N1)pdm09 in patients ≥65 years of age. IVE estimates for influenza B/Yamagata were homogenous (I^2^ = 0.0%).

**Conclusions:**

These results, which were based on data collected from the GIHSN during the 2012–2013 influenza season, showed that influenza vaccines provided low to moderate protection against hospital admission with laboratory-confirmed influenza in adults targeted for influenza vaccination. In this population, IVE estimates against A(H1N1)pdm09 were sensitive to age group and study site. Influenza vaccination was moderately effective in preventing admissions with influenza B/Yamagata for all sites and age groups.

## Introduction

Influenza vaccination is universally recommended for individuals at increased risk for complications, but the effectiveness of current licensed vaccines has not been clearly established [1], [2]. Observational field studies have shown substantial variability in influenza vaccine effectiveness (IVE) by season, strain, and age group [3]–[6]. In addition, many of these studies are underpowered for subgroup analyses, complicating estimates of IVE for individual risks. Furthermore, differences in study design and outcome measures limit the ability to compare results across studies, and the external validity of the results is weakened when the study population does not fully represent the different vaccination settings worldwide.

Several networks have been created to provide more representative and robust estimates of IVE[7]–[11]. These networks use a more standardized approach for data collection, analysis, and reporting of IVE, but most employ passive surveillance and therefore are highly dependent on reporting timeliness and completeness [12]–[14]. Also, few networks include surveillance of severe cases requiring hospitalization.

The Global Influenza Hospital Surveillance Network (GIHSN) was launched in 2012 to address growing awareness that influenza-related hospitalization is a significant burden that remains insufficiently characterized. The GIHSN is a partnership between industry and public health institutions that uses active surveillance and a common core protocol to collect data on the epidemiology of severe influenza, as defined by hospitalization with laboratory-confirmed influenza. The principal aim of the GIHSN is to estimate, when feasible, IVE against hospitalization with influenza. Data collection in the GIHSN is coordinated by regional centers. In the GIHSN’s first season (2012–2013), five coordinating centers covering 14 hospitals participated, including the *Centro Superior de Investigación en Salud Pública* (now FISABIO) (Valencia, Spain); the *Reseau National d’Investigation Clinique en Vaccinologie* (France), the Research Institute of Influenza (St. Petersburg, Russian Federation), the D.I. Ivanovsky Institute of Virology, Moscow, Russian Federation, and, as a pilot partner, the National Influenza Reference Laboratory (Cappa-Istanbul, Turkey).

Here, we used a test-negative approach [15], [16] to estimate IVE against hospitalization with laboratory-confirmed influenza. Validity of the pooled dataset was assessed by quantifying the heterogeneity in the effect estimates across the different study sites.

## Materials and Methods

### Study Design

This was a multi-center, prospective, active surveillance, hospital-based epidemiological study carried out during the 2012–2013 Northern Hemisphere influenza season. Data were collected from14 hospitals, including five located in Valencia, Spain (Hospital General de Castellon; Hospital de la Plana; Hospital Pesset; Hospital San Juan de Alicante; Hospital General de Elda), five in France (Cochin Hospital, Paris; Bichat Hospital, Paris; Limoges Hospital; St. Eloi Hospital, Montpellier; Lyon Hospital), and four in the Russian Federation (City Infectious Diseases Hospital #30, St. Petersburg; Children’s Infectious Hospital #5, St. Petersburg; Children’s City Hospital #4, St. Petersburg; Clinical Hospital for Infectious Diseases, Moscow). Hospitals in Turkey were not included because they were pilot partners at the time of this study. The principal objective was to estimate IVE against hospitalization with laboratory-confirmed influenza.

The protocol used by the GIHSN was approved by each site’s Ethics Research Committee: *Comité Ético de la Dirección General de Salud Pública y Centro Superior de Investigación en Salud Pública* (CEIC-DGSP-CSISP); *Comité de Protection des Personnes Ile-de-France III*; Ethic Committee of Hospital #1 for Infectious Diseases of Moscow Health Department; Ethics Committee of the Research Institute of Influenza, St. Petersburg; Istanbul University, Istanbul Faculty of Medicine, Ethical Committee for Clinical Research. All patients provided written informed consent. Briefly, data on hospitalized patients with a diagnosis possibly associated with influenza were collected by an active surveillance system composed of healthcare professionals trained to follow a generic study protocol, and influenza was confirmed by reverse transcription-polymerase chain reaction (RT-PCR). At each site, case identification was adapted to the specific local settings of the health care delivery system and type of hospital, although all sites used the same case criteria for definitive inclusion and, in all cases, the study was conducted over a period defined by the weeks with positive specimens for influenza (Table S1). The study was conducted according to Ethical Principles for Medical Research Involving Human Participants of the World Medical Association, the Declaration of Helsinki, and the International Ethical Guidelines for Epidemiological Studies.

### Study Population

Non-institutionalized adults that were residents of Valencia, Spain or who held a national social security affiliation (France) and were hospitalized for at least 24 h in one of the participating hospitals were considered for inclusion in the GIHSN database. Also, patients admitted at the emergency department (Valencia, France, Russian Federation) and at certain hospital wards (France, Russian Federation) were considered if they had pre-defined chief complaints presumably associated with a previous influenza infection [6]. After informed consent was obtained, patients were screened for the following inclusion criteria: onset of influenza-like-illness (ILI) within 7 days of admission to the hospital; influenza vaccination not contraindicated; not previously positive for influenza virus in the 2012–2013 season; and not hospitalized within 30 days of the current admission. ILI was defined as the presence of at least one systemic symptom (fever or feverishness, malaise, headache or myalgia) and at least one respiratory symptom (cough, sore throat or shortness of breath).

### Study Conduct

At enrollment, a nasopharyngeal and a pharyngeal swab were collected and patients were interviewed by a hospital physician, clinical research associate, or both (Russian Federation and France) or a dedicated study nurse (Valencia). Swabs were stored at −20°C. The following data were collected during the interview or by searching clinical records: demographic characteristics; anthropometric measures; information on the ILI episode; dates of symptom onset, hospitalization, and swabbing; antiviral treatment received; intense care unit admission; death during hospitalization; main hospital admission and discharge diagnostics; presence of chronic diseases; pregnancy status; number of hospital admissions in the past 12 months; number of general practitioner consultations in the previous 3 months; smoking habits; and vaccination against influenza in the current (2012–2013) and previous (2011–2012) seasons. Physicians involved in clinical care of patients were also involved in patient recruitment but were not involved in case ascertainment.

Social class was assigned according to occupation as described previously [17]. Functional status before ILI onset was ascertained in patients ≥65 years of age using the Barthel index [18] and categorized as follows: total dependence, 0–15; severe dependence, 20–35; mild to moderate dependence, 40–90; no dependence, ≥95. Vaccination status during the current season was ascertained from registries, vaccination cards, and interviews with patients, their families, and their physicians. Patients were considered vaccinated if they had received at least one dose of the 2012–2013 seasonal vaccine >14 days before the onset of ILI symptoms. Local vaccination policies and vaccines available at each coordinating site are summarized in Table S2.

### Laboratory Confirmation of Influenza

Commercially available (Russian Federation) or in-house (Valencia and France) RT-PCR assays were used to detect influenza A (subtypes H3 and H1) and influenza B (Yamagata and Victoria lineages) viruses in swabs (Text S1).

### Data Management, Calculations, and Statistical Analysis

Coordinating sites collected anonymized data and checked for missing, inconsistent, or incorrect data. Whenever possible, queries of any inconsistencies or missing data were resolved by the investigators at each of the study sites. Missing data were not replaced for the statistical analyses. Data from each coordinating site were shared with the network coordinating center (FISABIO, Valencia, Spain) through a secured web-based system.

Differences in the distribution of variables were estimated using a chi-square or T-test. A P-value of less than 0.05 was considered to indicate statistical significance.

The primary outcome measure was hospital admission with laboratory-confirmed influenza. Secondary outcome measures were hospital admissions with laboratory-confirmed influenza A(H1N1)pdm09, A(H3N2), or B/Yamagata.

IVE was determined in patients ≥18 years of age who had been swabbed within 7 days of the onset of ILI symptoms and who had been targeted for influenza vaccination because they were obese, pregnant, or ≥65 years of age, or had recorded comorbidities [19]. In addition, patients were excluded from IVE estimates and analysis if they had received a homeopathic vaccine. IVE was estimated as (1−odds ratio[OR]) ×100, where the OR compared the vaccine coverage rate between influenza-positive and influenza-negative patients. Records for which outcome, exposure, or confounding variables were missing were excluded from the multivariate IVE analyses. The adjusted IVE was estimated by logistic regression using a random effects model with study site as a shared parameter for the pooled analysis and including week of symptom onset as a continuous variable, and age group, sex, hospitalization in the previous 12 months, presence of chronic conditions, and smoking habits as potential confounding factors. Parameters not normally distributed were transformed prior to analysis. Polynomial fitting was used for non-linear relationships between week of symptom onset and influenza positivity. The nonlinear relationship between the week of symptom onset (independent variable) and influenza positivity (dependent variable) was modeled as an n^th^ order polynomial, yielding the general polynomial regression model y = β_0_+β_1_x +β_2×2_ +β_3×3_+…β_n_x_n_ + Σyz_i_ + µ_i_, where the expected value of a dependent variable y (log of the odds of either influenza positivity overall, H1N1, H2N3, B/Yamagata or B/Victoria) was modeled in terms of the value of the independent variable x (week of onset), β_n_ are the coefficients, Σyz_i_ are the effects of the covariates, and µ_i_ are the random effects representing between-site variability[20]. Sensitivity analysis was performed by including only samples taken within 4 days of symptom onset. A *P*-value <0.05 was considered to indicate statistical significance. Heterogeneity in IVE estimates was assessed using the I^2^ statistic [21]–[23]. Potential sources of heterogeneity, including coordinating site, age, and influenza subgroup were examined in ad-hoc analyses. Heterogeneity was defined as low if I^2^ statistic <25%, moderate if 25% to 49%, high if ≥50% as described previously[22].

Statistical analyses were performed using Stata version 13.1 (College Station, TX).

## Results

### Patients

A total of 9150 patients were screened by the 14 participating hospitals (Table 1). A total of 6581 patients met the criteria for inclusion in the GIHSN database. Of these, 2184 patients met criteria for and had available data for inclusion in the IVE analysis (896 in Valencia, 371 in France, 121 in St. Petersburg, and 670 in Moscow).

**Table 1:** Subjects included and excluded by coordinating site.

	Valencia		France		St Petersburg		Moscow		All	
Category	n	%	n	%	n	%	n	%	n	%
Eligible patients	5038	100.0	449	100.0	1986	100.0	1677	100.0	9150	100.0
Exclusions										
Nonresident	63	1.3	0	0.0	0	0.0	0	0.0	63	0.7
Institutionalized	329	6.5	0	0.0	7	0.4	21	1.3	357	3.9
Unable to communicate	250	5.0	0	0.0	47	2.4	2	0.1	299	3.3
Did not give consent	156	3.1	0	0.0	166	8.4	11	0.7	333	3.6
Did not meet the ILI case definition	1350	26.8	2	0.4	67	3.4	42	2.5	1461	16.0
>7 days between symptomonset and admission	485	9.6	2	0.4	3	0.2	63	3.8	553	6.0
Previous hospitalization <30 days ago	2	0.0	0	0.0	35	1.8	19	1.1	56	0.6
No swab taken	1	0.0	0	0.0	7	0.4	115	6.9	123	1.3
Specimen collected >7days after ILI onset	44	0.9	12	2.7	1	0.1	4	0.2	61	0.7
PCR result unavailableor sample inadequate	66	1.3	4	0.9	2	0.1	8	0.5	80	0.9
Outside of analysis period	785	15.6	0	0.0	51	2.6	3	0.2	839	9.2
Age <18 years	396	7.9	0	0.0	1169	58.9	358	21.3	1923	21.0
Previous influenza infection within the season	0	0.0	0	0.0	3	0.2	0	0.0	3	0.0
Contraindication for vaccination	0	0.0	0	0.0	9	0.5	2	0.1	11	0.1
Influenza vaccine status unknown/missing	0	0.0	2	0.4	4	0.2	7	0.4	13	0.1
Homeopathic vaccine given	0	0.0	18	4.0	0	0.0	0	0.0	18	0.2
Not belonging to population targeted for vaccination	89	1.8	38	8.5	294	14.8	352	21.0	773	8.4
Included in the analysis	1022	20.3	371	82.6	121	6.1	670	40.0	2184	23.9

The most important reasons for exclusion from IVE analysis were not meeting the ILI case definition(n = 1461), being hospitalized outside of the analysis period (n = 839), having more than 7 days between symptom onset and hospital admission (n = 553), aged under 18 years (n = 1923) and not belonging to population targeted for vaccination (n = 773). Most of the exclusions (4016 of 4667) were in Valencia and were due to the broad selection criteria, which were designed to capture the maximum number of patients hospitalized for reasons that have been or could be associated with influenza infection.

### Influenza Positives

Of the 2184 patients included, 675 (30.9%) tested positive for influenza by RT-PCR (Table 2). A(H1N1)pdm09 was the most frequently identified influenza virus (41.9%), followed by B/Yamagata (28.9%) and influenza A(H3N2) (15.1%).

**Table 2:** RT-PCR results at each site overall and in patients 18–64 and ≥65 years of age.

			RT-PCR result^a^				Influenza strain^b^											
			Negative		Positive		A(H1N1)pdm09		A(H3N2)		B/Yamagata)		B/Victoria		A untyped		B untyped	
Age group	Site	N	n	%	n	%	n	%	n	%	n	%	n	%	n	%	n	%
Overall	All	2184	1509	69.1	675	30.9	283	41.9	102	15.1	195	28.9	18	2.7	25	3.7	52	7.7
	Valencia	992	822	82.9	170	17.1	54	31.8	5	2.9	108	63.5	2	1.2	1	0.6	0	0.0
	France	371	249	67.1	122	32.9	24	19.7	38	31.1	49	40.2	3	2.5	8	6.6	0	0.0
	St. Petersburg	121	39	32.2	82	67.8	28	34.1	14	17.1	28	34.1	2	2.4	10	12.2	0	0.0
	Moscow	670	369	55.1	301	44.9	177	58.8	45	15.0	10	3.3	11	3.7	6	2.0	52	17.3
18–64 y	All	1065	632	59.3	433	40.7	229	52.9	63	14.5	60	13.9	15	3.5	20	4.6	46	10.6
	Valencia	206	169	82.0	37	18.0	19	51.4	1	2.7	16	43.2	1	2.7	0	0.0	0	0.0
	France	133	85	63.9	48	36.1	16	33.3	13	27.1	13	27.1	2	4.2	4	8.3	0	0.0
	St. Petersburg	93	28	30.1	65	69.9	23	35.4	8	12.3	23	35.4	1	1.5	10	15.4	0	0.0
	Moscow	633	350	55.3	283	44.7	171	60.4	41	14.5	8	2.8	11	3.9	6	2.1	46	16.3
≥65 y	All	1119	877	78.4	242	21.6	54	22.3	39	16.1	135	55.8	3	1.2	5	2.1	6	2.5
	Valencia	816	683	83.7	133	16.3	35	26.3	4	3.0	92	69.2	1	0.8	1	0.8	0	0.0
	France	238	164	68.9	74	31.1	8	10.8	25	33.8	36	48.6	1	1.4	4	5.4	0	0.0
	St. Petersburg	28	11	39.3	17	60.7	5	29.4	6	35.3	5	29.4	1	5.9	0	0.0	0	0.0
	Moscow	37	19	51.4	18	48.6	6	33.3	4	22.2	2	11.1	0	0.0	0	0.0	6	33.3

a Percentages are compared to the total of all patients in the category.

b Percentages are compared to influenza-positive patients.

#### Strains isolated at each site

**Figure 1:**
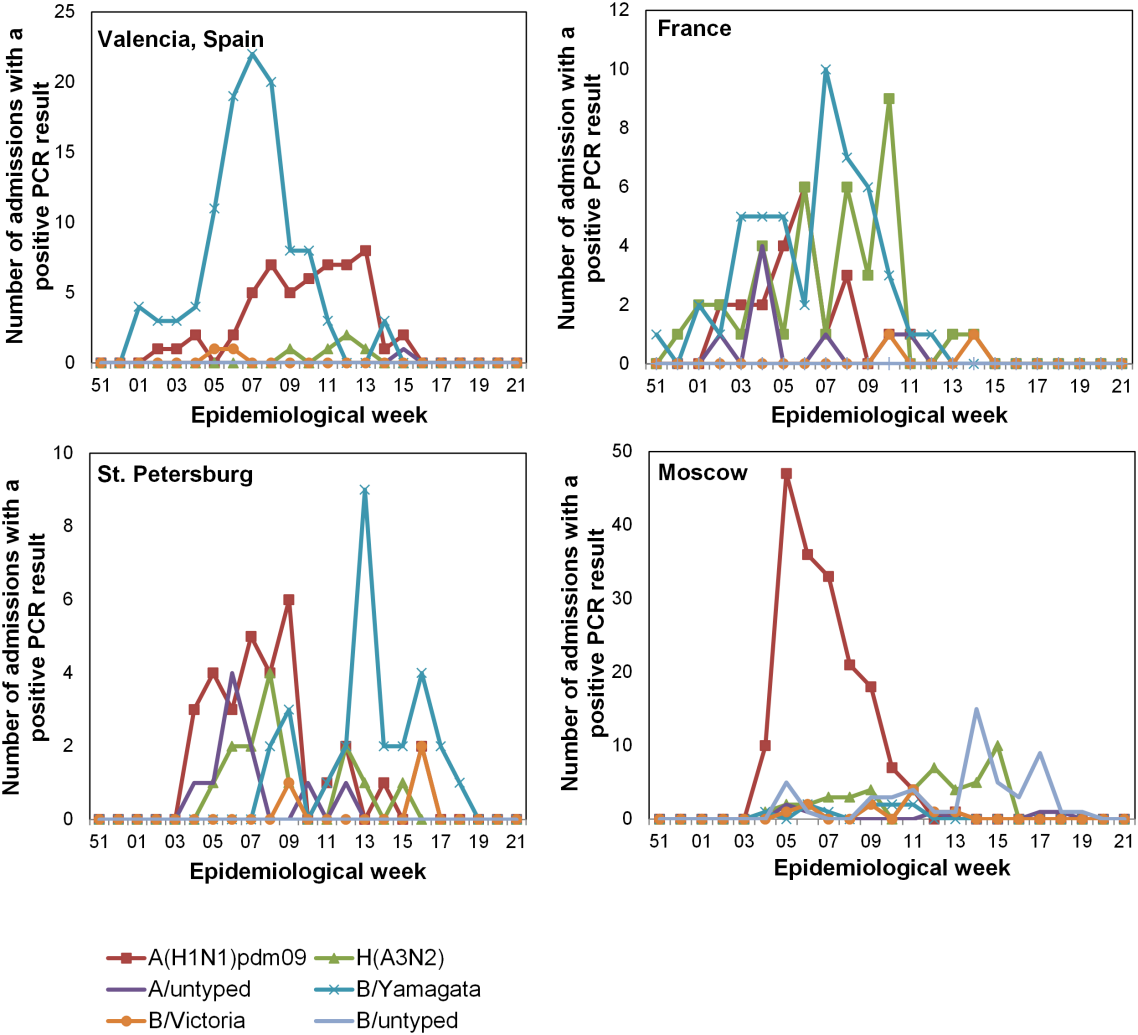
Number of admissions by epidemiological week at each site. The number of patients enrolled and included in the IVE analysis is shown by epidemiological week at each site for each influenza strain.

B/Yamagata was the predominant strain isolated from patients in Valencia (63.5% of isolates), while A(H1N1)pdm09 predominated in Moscow (58.8% of isolates). In St. Petersburg, A(H1N1)pdm09 and B/Yamagata predominated and were present at similar frequencies. In France, A(H3N2) and B/Yamagata predominated (Table 2 and Figure 1).

At each site, the distribution of strains in the patients changed as the season progressed (Figure 1). For example, in Valencia, B/Yamagata predominated early in the season, with a peak at epidemiological week 2013–7, whereas A(H1N1)pdm09 predominated later in the season, with a peak at epidemiological week 2013–13. In contrast, in St. Petersburg and Moscow, A(H1N1)pdm09 predominated early in the season, while B/Yamagata predominated later. The pattern in France was different than either of these countries, with several strains coexisting throughout the influenza season.

#### Strains isolated by age group

A(H1N1)pdm09 was more frequently isolated from patients <65 than ≥65 years of age (52.9% vs., 22.3%, p<0.001 by Chi^2^ test). B/Yamagata was more frequently isolated from patients ≥65 than <65 years of age (55.8% vs. 13.9%, p<0.001 by Chi^2^ test). A(H3N2) was evenly distributed among both age groups. B/Victoria was isolated from only 18 patients (2.7% overall) (Table 2).

### Patient Characteristics by Influenza Infection Status

Influenza-positive patients were younger than influenza-negative patients admitted to hospital (mean age, 51 vs. 63), less likely to be men, less likely to suffer from comorbidities, and less likely to have been hospitalized in the last year but more likely to have never smoked and more likely to have professional or non-manual skilled jobs (Table 3). Influenza-positive patients were less likely than influenza-negative patients to have been vaccinated for seasonal influenza during the 2012–2013 and 2011–2012 seasons. Of patients vaccinated for influenza during the 2012–2013 season, more influenza-negative patients had also received the 2011–2012 seasonal influenza vaccine.

**Table 3:** Characteristics of patients included in the IVE analysis by RT-PCR result.

		Influenza-negative	Influenza-positive	
Category	Subcategory	n (%)	n (%)	P Value
Total	-	1509 (69.1)	675 (30.9)	NC
Age	18–49 y	407 (27.0)	338 (50.1)	<0.001
	50–64 y	225 (14.9)	95 (14.1)	
	65–74 y	239 (15.8)	85 (12.6)	
	75–84 y	379 (25.1)	101 (15)	
	≥85 y	259 (17.2)	56 (8.3)	
Sex	Female	799 (53)	457 (67.7)	<0.001
	Male	710 (47)	218 (32.3)	
Comorbidities	0	376 (24.9)	297 (44)	<0.001
	1	512 (33.9)	219 (32.4)	
	≥2	621 (41.2)	159 (23.6)	
Obese (body massindex ≥30)	Yes	427 (28.3)	163 (24.2)	0.115
Hospitalized inthe last 12 months^a^	Yes	480 (32)	126 (18.8)	<0.001
General practitionervisits last 3 months^b^	0	567 (37.6)	376 (55.7)	<0.001
	1	318 (21.1)	117(17.3)	
	≥2	575 (38.1)	155(23)	
Smoking	Never	725 (48.1)	385 (57)	<0.001
	Past	525 (34.8)	168 (24.9)	
	Current	259 (17.2)	122 (18.1)	
Socioeconomicclass	Professional tonon-manual-skilled	401 (26.6)	256 (37.9)	<0.001
	Manual-skilled	178 (11.8)	67 (9.9)	
	Manual-non-skilled	638 (42.3)	143 (21.2)	
	Unknown	292 (19.4)	209 (31)	
Functionalcapacity	No impairment^c^^,^ d	536 (61.2)	146 (60.3)	<0.001
Influenzavaccination^e^	2012–2013	641 (42.5)	132 (19.6)	<0.001
Influenza vaccinationbased on medicalrecords only	2012–2013	584(38.7)	112(16.6)	<0.001
	2011–2012^f^	638 (42.3)	152 (22.5)	<0.001
Time from onset ofsymptoms to swabbing	1 to 2 d	448 (29.7)	260 (38.5)	<0.001
	3 to 4 d	641 (42.5)	283 (41.9)	
	5 to 7 d	420 (27.8)	132 (19.6)	

*P*-values were determined by Pearson’s chi-square test. NC, not calculated.

a N = 2158.

b N = 2032.

c N = 1069.

d No impairment defined as a Barthel score >60.

e Data on vaccination were exclusively from self-reporting for only 5.2% of all vaccinated patients. None of the patients with clinical records of vaccination self-reported not having been vaccinated.

f N = 2168.

The mean interval between symptom onset and specimen collection was similar for influenza-positive and influenza-negative patients (mean ± standard deviation = 3.1±1.6vs. 3.5±1.7 days), although more influenza-positive than influenza-negative patients were swabbed within 2 days. The risk of being influenza positive decreased by 3% (95% CI, 2% to 4%) (P for trend <0.0001) for each day elapsed between symptom onset and swabbing.

### Patient Characteristics by Vaccination Status

Patients vaccinated during the year of the study (2012–2013) were older than unvaccinated patients (mean, 76 vs. 50 y) (Table 4). Vaccinated patients were also more likely to be men, suffer from chronic conditions, to have been hospitalized in the last year, to have visited the general practitioner in the last 3 months, to be past smokers, and to have been influenza-vaccinated the previous year (2011–2012).

**Table 4:** Characteristics of patients included in the IVE analysis according to vaccination the current year (2012–2013).

		Notvaccinated	Vaccinated	
Category	Subcategory	n (%)	N (%)	*P*-value
Total		1411 (64.6)	773 (35.4)	NC
Age group	18–49 y	713 (50.5)	32 (4.1)	<0.001
	50–64 y	236 (16.7)	84 (10.9)	
	65–74 y	171 (12.1)	153 (19.8)	
	75–84 y	185 (13.1)	295 (38.2)	
	≥85 y	106 (7.5)	209 (27)	
Sex	Female	944 (66.9)	312 (40.4)	<0.001
	Male	467 (33.1)	461 (59.6)	
Comorbidities	0	605 (42.9)	68 (8.8)	<0.001
	1	460 (32.6)	271 (35.1)	
	≥2	346 (24.5)	434 (56.1)	
Obese (body massindex ≥30)^a^	Yes	367 (26)	223 (29)	0.244
Hospitalized in thelast 12 months^a^	Yes	298 (21.3)	308 (40)	<0.001
General practitionervisits in the last 3 months^b^	0	809 (57.3)	134 (17.3)	<0.001
	1	238 (16.9)	197 (25.5)	
	≥2	313 (22.2)	417 (54)	
Smoking	Never	754 (53.4)	356 (46.1)	<0.001
	Past smoker	372 (26.4)	321 (41.5)	
	Current smoker	285 (20.2)	96 (12.4)	
Socioeconomicclass	Professional tonon-manual-skilled	573 (40.6)	84 (10.9)	<0.001
	Manual-skilled	171 (12.1)	74 (9.6)	
	Manual-unskilled	356 (25.2)	425 (55)	
	Unknown	311 (22)	190 (24.6)	
Functional capacity	No impairment^c^^,^ d	260 (56.3)	422 (64.2)	<0.001
Influenza vaccine	Previousseason (2011–2012)^e^	130 (9.2)	660 (85.4)	<0.001

*P*-values were determined by Pearson’s chi-square test. NC, not calculated.

a N = 2158.

b N = 2032.

c N = 1069.

d No impairment defined as a Barthel score >60.

e N = 2168.

### Patient Characteristics by Study Site

Patients in St. Petersburg (76.9%) and Moscow (94.5%) were mostly <65 years of age and had either no or one chronic disease (Table 5), regardless of influenza infection status (Table S3). In Moscow, 72.1% (483/670) of the patients were pregnant women (mean age, 28±5 years). The patients in France and Spain were evenly spread across age groups, and at least 70% suffered from one or more chronic condition. The pattern of chronic conditions was similar in Valencia and France (cardiovascular disease, chronic obstructive pulmonary disease, and diabetes), whereas in Moscow and St. Petersburg, the main chronic illness reported was cardiovascular disease. The median (interquartile range) number of chronic illnesses in patients with comorbidities was 1 (1–2) in Valencia, 2 (1–2) in France, 1 (1–1) in St. Petersburg, and 0 (0–1) Moscow. Influenza vaccine uptake was low in Moscow (3.3%) and St. Petersburg (0.8%) but moderate in Valencia (55.4%) and France (53.4%).

**Table 5:** Characteristics of patients included in the IVE analysis at each site.

		Valencia (N = 1022)	St. Petersburg (N = 121)	Moscow (N = 670)	France (N = 371)	Overall (N = 2184)
Category	Subcategory	n (%)	n (%)	n (%)	n (%)	n (%)
Age group	18–49 y	66 (6.5)	37 (30.6)	578 (86.3)	64 (17.3)	745(34.1)
	50–64 y	140 (13.7)	56 (46.3)	55 (8.2)	69 (18.6)	320 (14.7)
	65–74 y	225 (22.0)	14 (11.6)	20(3.0)	65 (17.5)	324 (14.8)
	75–84 y	359 (35.1)	12 (9.9)	14 (2.1)	95 (25.6)	480 (22.0)
	≥85 y	232 (22.7)	2 (1.7)	3 (0.5)	78 (21.0)	315 (14.4)
Sex	Male	559 (54.7)	52 (43.0)	121 (18.1)	196 (52.8)	928 (42.5)
	Female	463 (45.3)	69 (57.0)	549 (81.9)	175 (47.2)	1256 (57.5)
Comorbidities	0	135 (13.2)	27 (22.3)	465 (69.4)	46 (12.4)	673 (30.8)
	1	378 (37.0)	67 (55.4)	159 (23.7)	127 (34.2)	731 (33.5)
	≥2	509 (49.8)	27 (22.3)	46 (6.9)	198 (53.4)	780 (35.7)
Hospitalized inthe last 12 months^a^	Yes	370 (36.2)	13 (11.9)	54 (8.1)	169 (45.6)	606(27.8)
General practitionervisits last three months^b^	0	264 (25.8)	75 (62.0)	603 (90.0)	1 (0.3)	943 (43.2)
	1	266 (26.0)	23 (19.0)	37 (5.5)	109 (29.4)	435 (19.9)
	≥2	492 (48.1)	12 (10.0)	30 (4.3)	196 (52.8)	730 (33.42)
Smoking	Never	462 (45.2)	86 (71.1)	391 (58.4)	171 (46.1)	1110 (50.8)
	Pastsmoker	389 (38.1)	4 (3.3)	183 (27.3)	117 (31.5)	693 (31.7)
	Currentsmoker	171 (16.7)	31 (25.6)	96 (14.3)	83 (22.4)	381 (17.5)
Time from onsetof symptoms toswabbing	1 to 2 d	230 (22.5)	36 (29.8)	337 (50.3)	105 (28.3)	708 (32.4)
	3 to 4 d	483 (47.3)	56 (46.3)	233 (34.8)	152 (41.0)	924 (42.3)
	5 to 7 d	309 (30.2)	29 (24.0)	100 (14.9)	114 (30.7)	552 (25.3)
Disability^c^	NoImpairment	52 (63.9)	6 (21.4)	0 (0)	155 (65.1)	682 (61)
Vaccinated forinfluenza duringthe current season (2012–2013)	Yes	566 (55.4)	4 (3.3)	5 (0.8)	198 (53.4)	773 (35.4)

a Missing: St. Petersburg, n = 12; France, n = 1.

b Missing: St. Petersburg, n = 11; France, n = 65.

c Presented only for patients ≥65 years of age. No impairment defined as a Barthel score >60. Data missing: St. Petersburg, n = 13; Moscow, n = 37.

### IVE

#### Overall

Influenza-positive patients were less likely to have been vaccinated during the year of the study (2012–2013) than influenza-negative patients (adjusted OR = 0.67 [95% CI, 0.51 to 0.89]; P = 0.0060). This corresponded to an overall adjusted IVE of 33% (95% CI, 11% to 49%) (Table 6). IVE was similar in patients <65 and ≥65 years of age (35% [95% CI, −15% to 63%] vs. 31% [95% CI, 4% to 51%]). When pregnant women were excluded, values were similar (IVE [95% CI] = 33% [10% to 49%] overall, 15% [−23% to 54%] for A(H1N1), 33% [−32% to 66%]for A(H3N2), and 42%[16% to 60%]for B/Yamagata; data not shown). IVE estimates in subjects ≥65 years of age were similar when adjusted for disability (i.e. Barthel score as a categorical variable) (data not shown).

**Table 6:** Pooled IVE in hospitalized patients swabbed within 7 days of symptom onset.

		Influenzapositive			Influenzanegative			IVE adjusted forsite^a^			Fully adjustedIVE^a^^,^ b		
Influenzastrain	Agegroup	n	N	%	N	N	%	IVE	95% CI		IVE	95% CI	
Allinfluenza	Overall	132	675	20%	641	1509	42%	44%	27%	57%	33%	11%	49%
	<65 y	21	433	5%	95	632	15%	51%	15%	72%	35%	−15%	63%
	≥65 y	111	242	46%	546	877	62%	43%	22%	58%	31%	4%	51%
A(H1N1)	Overall	33	286	12%	641	1509	42%	52%	25%	70%	23%	−26%	53%
	<65 y	8	232	3%	95	632	15%	59%	7%	82%	40%	−43%	75%
	≥65 y	25	54	46%	546	877	62%	34%	−22%	64%	13%	−68%	55%
A(H3N2)	Overall	22	102	22%	641	1509	42%	33%	−23%	64%	30%	−37%	64%
	<65 y	5	63	8%	95	632	15%	18%	−147%	73%	19%	−162%	75%
	≥65 y	17	39	44%	546	877	62%	46%	−16%	75%	39%	−40%	73%
B/Yamagata	Overall	70	195	36%	641	1509	42%	43%	20%	59%	43%	17%	60%
	<65 y	6	60	10%	95	632	15%	58%	−5%	83%	52%	−25%	81%
	≥65 y	64	135	47%	546	877	62%	46%	23%	63%	41%	12%	61%

CI, confidence interval; IVE, influenza vaccine effectiveness.

a Site as a random effect.

b Adjusted by week of symptom onset, age group, sex, hospitalization in the previous 12 months, presence of chronic conditions, and smoking habits.

#### By strain

IVE estimates were 23% (95% CI, −26% to 53%) for influenza A(H1N1)pdm09, 30% (95% CI, −37% to 64%) for influenza A(H3N2), and 43% (95% CI, 17% to 60%) for influenza B/Yamagata(Table 6). IVE was higher in patients <65 years of age than in those ≥65 years of age for A(H1N1)pdm09 (40% [95% CI, −43% to 75%] vs. 13% [−68% to 55%]) and B/Yamagata (52% [95% CI, −25% to 81%] vs. 41% [95% CI, 12% to 61%]). Results were similar when the analyses were restricted to patients swabbed within 4 days of symptom onset (Table S4). Significant adjusted IVE estimates were obtained for all influenza in subjects ≥65 years of age and the B/Yamagata lineage, also in subjects ≥65 years of age. Analysis of the influence of socioeconomic factors was not possible due to a high proportion of “don’t know” responses (data not shown).

### Heterogeneity in IVE Estimates

**Figure 2:**
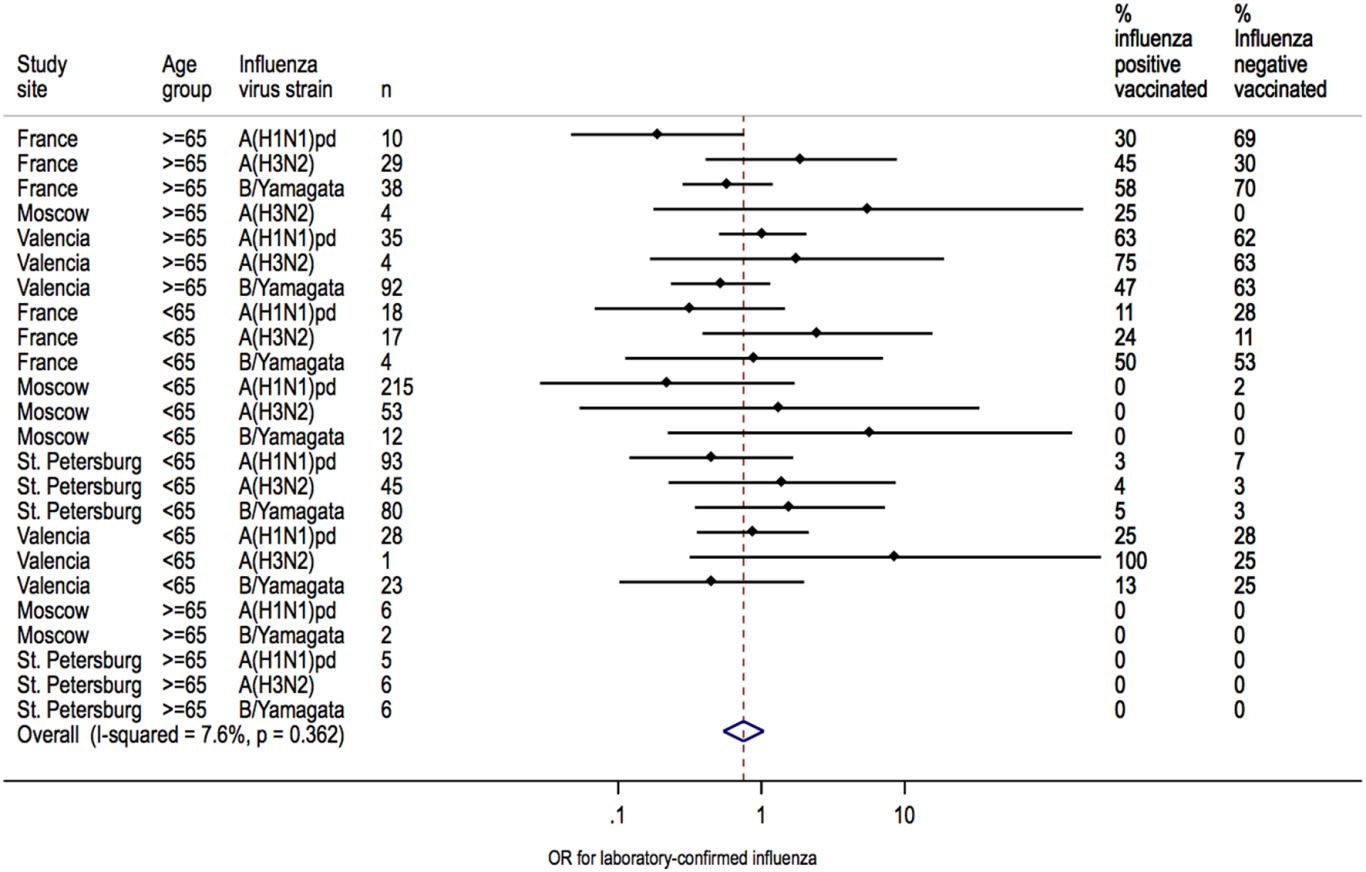
Heterogeneity of IVE estimates at each site overall.

**Figure 3:**
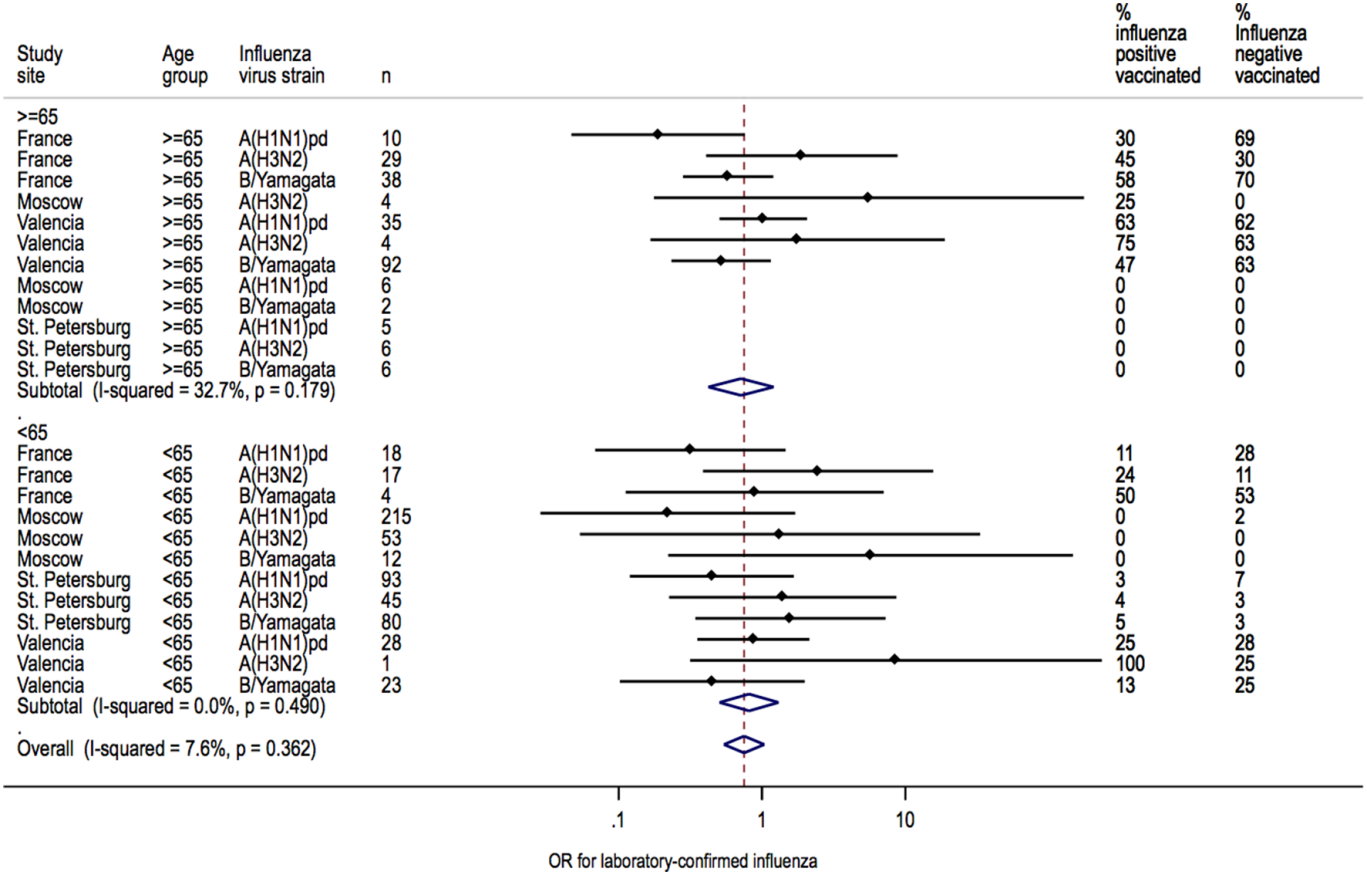
Heterogeneity of IVE estimates at each site for each age group.

**Figure 4:**
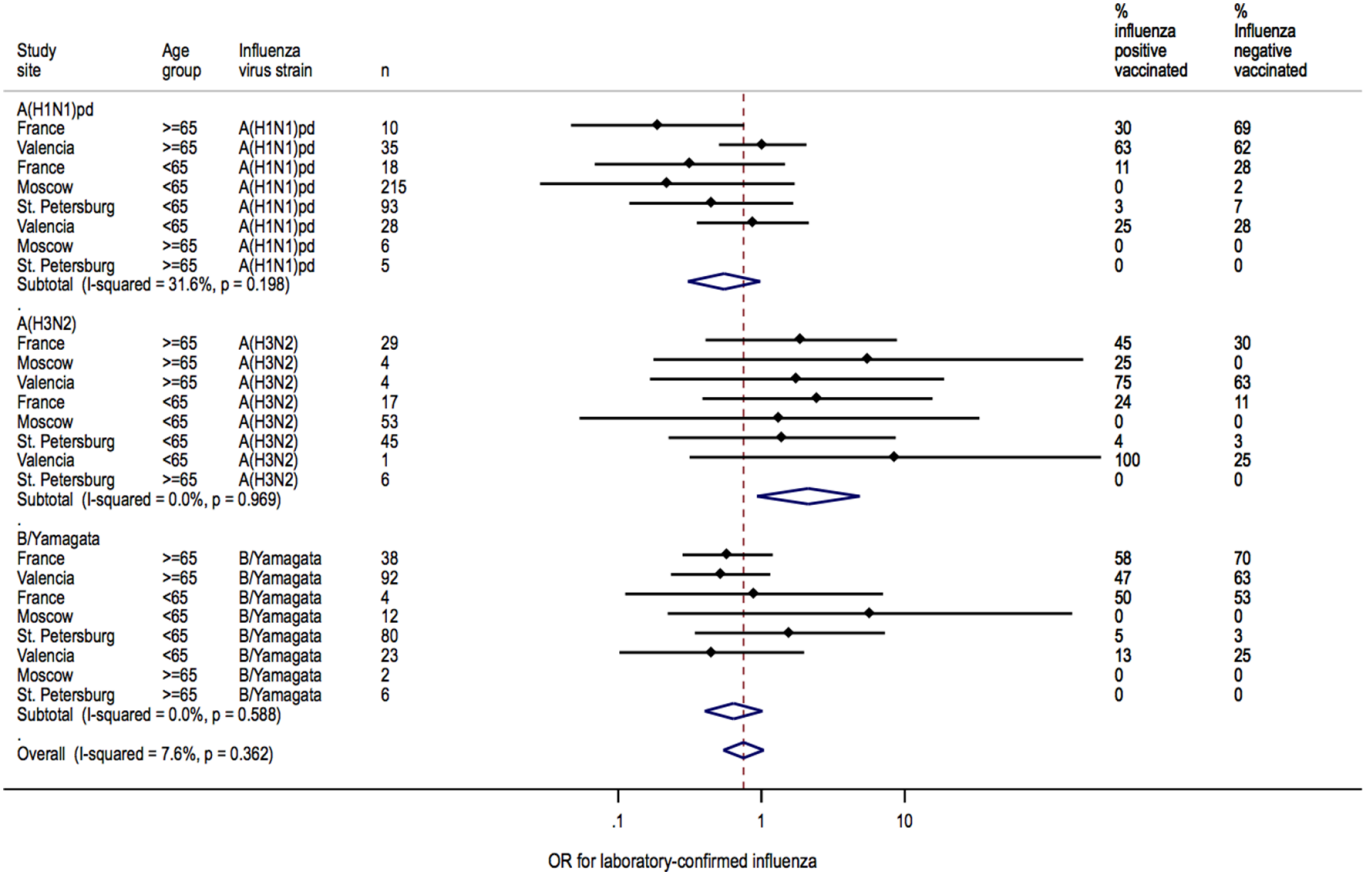
Heterogeneity of IVE estimates at each site for each strain.

Heterogeneity between sites was low for overall IVE estimates (I^2^ = 7.6%; *P* = 0.362) (Figure 2). Heterogeneity between sites was also low for IVE estimates in patients <65 years of age (I^2^ = 7.6%; *P* = 0.362) but moderate in patients ≥65 years of age (I^2^ = 32.7%; *P* = 0.179) (Figure 3). Heterogeneity across sites in IVE estimates was moderate for A(H1N1)pdm09 (I^2^ = 31.6%; *P* = 0.198), whereas IVE estimates for each site were homogenous for A(H3N2) (I^2^ = 0.0%, *P* = 0.969) and B/Yamagata (I^2^ = 0.0%; *P* = 0.588, respectively) (Figure 4).

**Figure 5:**
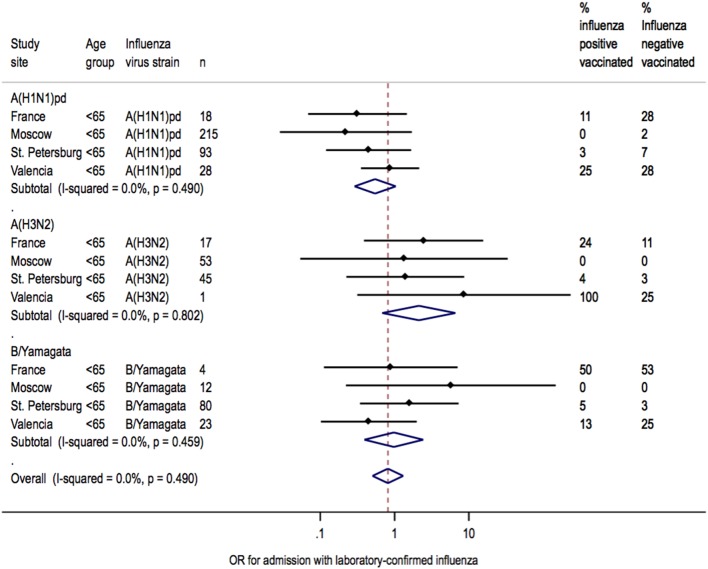
Heterogeneity of IVE estimates for each strain in patients 18–64 years of age.

**Figure 6:**
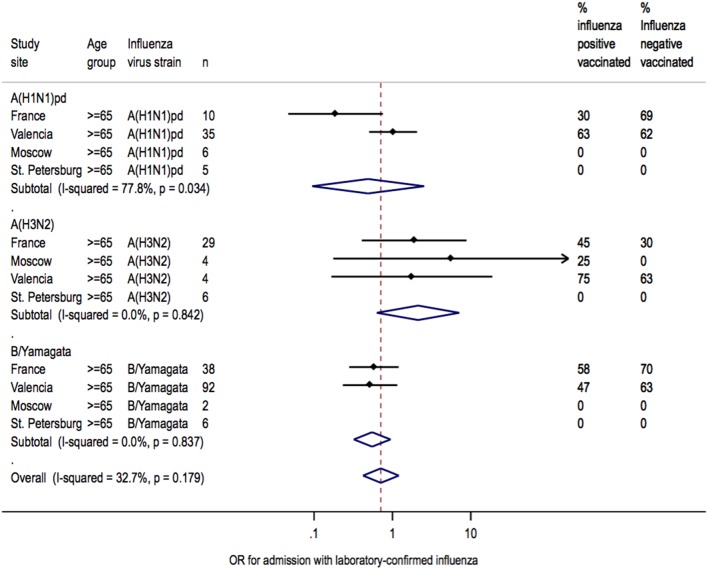
Heterogeneity of IVE estimates for each strain in patients ≥65 years of age.

IVE estimates against H3N2 or B/Yamagata were also homogenous when assessed by age group, strain, and study site (I^2^ = 0.0%; *P*>0.8) (Figures 5 and 6). Heterogeneity in IVE estimates was high (I^2^ = 78%; *P* = 0.0340) for A(H1N1)pdm09 in patients ≥65 years of age, with poorer protection in Valencia than in France.

All heterogeneity results were similar when assessed using adjusted IVE estimates (Figures S1, S2, and S3).

## Discussion

This study, performed in three different countries during the 2012–2013 influenza season, used a test-negative design to estimate IVE against hospitalization with laboratory-confirmed influenza in adults targeted for vaccination. All patients included in the IVE estimates and analysis had to have been tested for influenza within 7 days of the onset of ILI symptoms. The pooled adjusted IVE was 33% (95% CI, 11% to 49%) against hospitalization. Estimates of IVE for preventing hospital admissions were consistent and moderate across sites and age groups for influenza B/Yamagata (43% [95% CI, 17% to 60%]) but low and non-significant for influenza A(H1N1)pdm09 (30% [95% CI, −37% to 64%]) and A(H3N2) (23% [95% CI, −26% to 53%]). IVE estimates for A(H1N1)pdm09 were highly heterogeneous across study sites in patients ≥65 years of age but not in younger patients. Influenza A(H1N1)pdm09 and B/Yamagata followed by A(H3N2) were the most common strains isolated. These results agree with other interim and preliminary results published for the 2012–2013 influenza season [3], [5], [24]–[29].

The low IVE estimates in this study might have been due to genetic drift in influenza at key antigenic sites[5]. Genetic and possible antigenic mismatches have been described in Europe for A(H1N1)pdm09 and A(H3N2)[23]–[25]. Vaccines for the 2012–2013 season containing A/Victoria/361/2011 antigens have been reported to induce antibodies in humans that bind less effectively to most cell-propagated influenza A(H3N2), apparently due to antigenic changes in earlier A/Victoria/361/2011-like vaccine viruses associated with adaptation of the virus to propagation in eggs[30]. Accordingly, vaccines for the 2013–2014 northern hemisphere season are recommended to contain A(H3N2) virus that is antigenically like the cell-propagated prototype virus A/Victoria/361/2011[30]. In contrast, in two preliminary analyses of North American data, IVE was moderate and significant against A(H3N2), although this was associated with a good antigenic match between circulating and vaccine A(H3N2) strains [7], [31].

The IVE estimates in this study were similar to those reported in sentinel hospital-based studies [32], [33] but were lower than reported for general practitioner-attended influenza outcomes[26], [29]. This might be because of different effectiveness for different clinical outcomes or because of the generally older age and poorer health of patients requiring hospital admission for influenza infections. Indeed, our study patients were, on average, older and in poorer health than those in the general practitioner sentinel studies. Also, in contrast to some of these general practitioner sentinel studies, our estimates of IVE against influenza A strains were similar across age groups. However, in all reports, including ours, IVE against B/Yamagata influenza was moderate, despite differences in baseline patient characteristics.

The validity of IVE estimates can be influenced by nonspecific case definition, ascertainment, information bias and confounding. To overcome some of these limitations, we used a hospital-based active-surveillance approach to identify eligible patients. Despite each site adapting screening criteria to the particular circumstances of their health care systems and the participating hospitals, all sites consistently applied the network eligibility criteria. In addition, to reduce bias, at all sites, subjects were screened and included in the study without previous knowledge of their exposure or outcome status and belonged to the same population at risk for influenza infection, namely, those targeted for vaccination[34]. All sites used the common GIHSN core protocol and close follow-up and feedback between the coordination center and the different sites to ensure that standard procedures and monitoring were employed throughout the influenza season.

We used a highly specific outcome definition of severe influenza, with influenza infection confirmed by RT-PCR performed in highly qualified central laboratories. To minimize the impact of false negatives on IVE estimates, we excluded patients swabbed more than 7 days after the onset of symptoms [34]. IVE was estimated using the widely used test-negative approach, which has been shown to give consistent results[15], and the analysis of IVE was restricted to periods with similar influenza circulation patterns[16], [35]. Furthermore, the IVE was calculated on the basis of ORs determined using a random effects model, which allowed us to take into account potential differences, including type of vaccine, vaccination programs, the levels of immunity across different population and settings, and different use of hospital emergency departments [36], [37].

Underlying heterogeneity across study sites may have compromised the accuracy of the overall IVE estimates. We observed high heterogeneity in the estimates of IVE against A(H1N1)pdm09 by site in patients ≥65 years of age. This was mainly due to opposing directions of IVE estimates in France and Valencia. Identifying the host and pathogen factors that may have contributed to this variability is complicated by limited understanding of the factors that affect annual IVE estimates [38]. One possibility for the heterogeneity is site-specific genetic and antigenic differences between circulating A(H1N1)pdm09 and seasonal vaccine viruses[25], [39], [40]. We cannot exclude the possibility that the differences between sites are due, at least to some extent, to different vaccines being used.

The heterogeneity of pooled analyses from existing influenza networks and the relevance of IVE estimates across sites sharing a core standardized protocol remain largely unknown [41], [42]. A thorough assessment and exploration of the heterogeneity inherent to multicenter studies is needed to evaluate the robustness of pooled IVE results and the identification of risk factors. One possible framework for understanding the heterogeneity of observational IVE data and how to interpret it is that provided by Beyer et al. who re-analyzed data from a 2010 Cochrane meta-analysis of IVE in the elderly[43]. By rearranging the data according to “a biological and conceptual framework based on the basic sequence of events throughout the ‘patient journey’”, they found a mean IVE against complications of 28% (95% CI, 26% to 30%) and against laboratory-confirmed disease of 49% (95% CI, 33% to 62%). They concluded that their findings provide “substantial evidence for the ability of influenza vaccine to reduce the risk of influenza infection and influenza-related disease and death in the elderly.” Although both their and our analyses were based on heterogeneous source data, we had similar findings and reached similar conclusions on the effectiveness of influenza vaccines.

The wide confidence intervals observed in our study suggests that small sample sizes may have compromised the precision around risk-specific IVE estimates and the power of statistical tests to detect all sources of heterogeneity. Therefore, random error could have affected our estimates. Accordingly, the IVE estimates should be interpreted with caution. The results of this study support the feasibility of estimating IVE against hospitalization for laboratory-confirmed influenza based on a global active-surveillance hospital-based network. New sites in China and Brazil, and a fully operational site in Turkey will be joining the GIHSN in the 2013–2014 season. This will increase its geographical representativeness and sample size, which will improve the validity and accuracy of data on influenza vaccine effects and their variability. This is especially important for attaining the principal public health objectives of preventing morbidity and premature mortality in people at high risk for complications from influenza.

## Supporting Information

Figure S1 **Heterogeneity in adjusted IVE estimates by age group.**
(TIF)

Figure S2 **Heterogeneity in adjusted IVE estimates at each site by strain.**
(TIF)

Figure S3 **Heterogeneity in adjusted IVE estimates by site.**
(TIF)

Table S1 **Hospital characteristics, inclusion and exclusion criteria, and influenza season definition for each site.**
(DOC)

Table S2 **Vaccination policies and vaccines available at each coordinating site.**
(DOC)

Table S3 **Characteristics of patients included in the IVE analysis by site and influenza infection status.**
(DOC)

Table S4 **Pooled IVE in hospitalized patients swabbed within 4 days of symptom onset.**
(DOC)

Table S5 **Diagnosis codes used to identify emergency admissions possibly associated with an influenza infection and considered for inclusion.**
(DOC)

Text S1 **GIHSN laboratory characteristics and procedures.**
(DOCX)

## References

[1] Monto AS, Ansaldi F, Aspinall R, McElhaney JE, Montano LF, et al (2009) Influenza control in the 21st century: Optimizing protection of older adults. Vaccine 27: 5043–5053. 19559118 10.1016/j.vaccine.2009.06.032

[2] Osterholm MT, Kelley NS, Sommer A, Belongia EA (2012) Efficacy and effectiveness of influenza vaccines: a systematic review and meta-analysis. Lancet Infect Dis 12: 36–44. 22032844 10.1016/S1473-3099(11)70295-X

[3] Bragstad K, Emborg H, Fischer TK, Voldstedlund M, Gubbels S, et al.. (2013) Low vaccine effectiveness against influenza A(H3N2) virus among elderly people in Denmark in 2012/13–a rapid epidemiological and virological assessment. Euro Surveill 18. 23410258

[4] Kelly HA, Sullivan SG, Grant KA, Fielding JE (2013) Moderate influenza vaccine effectiveness with variable effectiveness by match between circulating and vaccine strains in Australian adults aged 20–64 years, 2007–2011. Influenza Other Respi Viruses 7: 729–737. 10.1111/irv.12018 PMC5781205 23078073

[5] McMenamin J, Andrews N, Robertson C, Fleming D, Durnall H, et al.. (2013) Effectiveness of seasonal 2012/13 vaccine in preventing laboratory-confirmed influenza infection in primary care in the United Kingdom: mid-season analysis 2012/13. Euro Surveill 18. 10.2807/ese.18.05.20393-en 23399421

[6] Puig-Barbera J, Diez-Domingo J, Arnedo-Pena A, Ruiz-Garcia M, Perez-Vilar S, et al (2012) Effectiveness of the 2010–2011 seasonal influenza vaccine in preventing confirmed influenza hospitalizations in adults: a case-case comparison, case-control study. Vaccine 30: 5714–5720. 22819720 10.1016/j.vaccine.2012.07.006 PMC7115682

[7] Centers for Disease Control and Prevention (2013) Early estimates of seasonal influenza vaccine effectiveness–United States, January 2013. MMWR Morb Mortal Wkly Rep 62: 32–35. 23325354 PMC4604840

[8] Fielding JE, Grant KA, Garcia K, Kelly HA (2011) Effectiveness of seasonal influenza vaccine against pandemic (H1N1) 2009 virus, Australia, 2010. Emerg Infect Dis 17: 1181–1187. 21762570 10.3201/eid1707.101959 PMC3381383

[9] Rondy M, Puig-Barbera J, Launay O, Duval X, Castilla J, et al (2013) 2011–12 seasonal influenza vaccines effectiveness against confirmed A(H3N2) influenza hospitalisation: pooled analysis from a European network of hospitals. A pilot study. PLoS One 8: e59681. 23565159 10.1371/journal.pone.0059681 PMC3614550

[10] Skowronski DM, Janjua NZ, De Serres G, Winter AL, Dickinson JA, et al (2012) A sentinel platform to evaluate influenza vaccine effectiveness and new variant circulation, Canada 2010–2011 season. Clin Infect Dis 55: 332–342. 22539661 10.1093/cid/cis431

[11] Valenciano M, Ciancio B, team IMs (2012) I-MOVE: a European network to measure the effectiveness of influenza vaccines. Euro Surveill 17. 10.2807/ese.17.39.20281-en 23041022

[12] Brammer L, Budd A, Cox N (2009) Seasonal and pandemic influenza surveillance considerations for constructing multicomponent systems. Influenza Other Respi Viruses 3: 51–58. 10.1111/j.1750-2659.2009.00077.x PMC4634524 19496841

[13] Grijalva CG, Weinberg GA, Bennett NM, Staat MA, Craig AS, et al (2007) Estimating the undetected burden of influenza hospitalizations in children. Epidemiol Infect 135: 951–958. 17156502 10.1017/S095026880600762X PMC2870647

[14] Iwane MK, Edwards KM, Szilagyi PG, Walker FJ, Griffin MR, et al (2004) Population-based surveillance for hospitalizations associated with respiratory syncytial virus, influenza virus, and parainfluenza viruses among young children. Pediatrics 113: 1758–1764. 15173503 10.1542/peds.113.6.1758

[15] De Serres G, Skowronski DM, Wu XW, Ambrose CS (2013) The test-negative design: validity, accuracy and precision of vaccine efficacy estimates compared to the gold standard of randomised placebo-controlled clinical trials. Euro Surveill 18: pii = 20585. 10.2807/1560-7917.es2013.18.37.20585 24079398

[16] Foppa IM, Haber M, Ferdinands JM, Shay DK (2013) The case test-negative design for studies of the effectiveness of influenza vaccine. Vaccine 31: 3104–3109. 23624093 10.1016/j.vaccine.2013.04.026

[17] Galobardes B, Shaw M, Lawlor DA, Lynch JW, Davey Smith G (2006) Indicators of socioeconomic position (part 1). J Epidemiol Community Health 60: 7–12. 10.1136/jech.2004.023531 PMC2465546 16361448

[18] Mahoney FI, Barthel DW (1965) Functional Evaluation: The Barthel Index. Md State Med J 14: 61–65. 14258950

[19] [No authors listed] (2012) Vaccines against influenza WHO position paper - November 2012. Wkly Epidemiol Rec 87: 461–476. 23210147

[20] Motulsky A, Chritsopoulos A (2004) Fitting models to biological data using linear and non-linear regression: a practical guide to curve fitting. Oxford, UK: Oxford University Press.

[21] Higgins JP, Thompson SG (2002) Quantifying heterogeneity in a meta-analysis. Stat Med 21: 1539–1558. 12111919 10.1002/sim.1186

[22] Higgins JP, Thompson SG, Deeks JJ, Altman DG (2003) Measuring inconsistency in meta-analyses. BMJ 327: 557–560. 12958120 10.1136/bmj.327.7414.557 PMC192859

[23] Sterne JAC (2009) Meta-analysis in Stata: an updated collection from the Stata journal. College Station, TX: Stata Press.

[24] [No authors listed] (2012) Review of the 2011–2012 winter influenza season, northern hemisphere. Wkly Epidemiol Rec 87: 233–240. 22715518

[25] [No authors listed] (2013) Recommended composition of influenza virus vaccines for use in the 2014 southern hemisphere influenza season. Wkly Epidemiol Rec 88: 437–448. 24159667

[26] Castilla J, Martinez-Baz I, Martinez-Artola V, Fernandez-Alonso M, Reina G, et al (2013) Early estimates of influenza vaccine effectiveness in Navarre, Spain: 2012/13 mid-season analysis. Euro Surveill 18: 2. 23449182

[27] Centers for Disease Control and Prevention (2013) Interim adjusted estimates of seasonal influenza vaccine effectiveness - United States, February 2013. MMWR Morb Mortal Wkly Rep 62: 119–123. 23425960 PMC4604884

[28] Eick-Cost AA, Hu Z, Cooper MJ, Sanchez JL, Radin JM, et al (2013) Mid-season influenza vaccine effectiveness for the 2012–2013 influenza season. MSMR 20: 15–16. 23550929

[29] Valenciano M, Kissling E (2013) Team IMC-CS (2013) Early estimates of seasonal influenza vaccine effectiveness in Europe: results from the I-MOVE multicentre case-control study, 2012/13. Euro Surveill 18: 3. 23449183

[30] World Health Organization (2013) Recommended composition of influenza virus vaccines for use in the 2013–2014 northern hemisphere influenza season. Wkly Epidemiol Rec 88: 101–114. 23544236

[31] Skowronski DM, Janjua NZ, De Serres G, Dickinson JA, Winter AL, et al.. (2013) Interim estimates of influenza vaccine effectiveness in 2012/13 from Canada's sentinel surveillance network, January 2013. Euro Surveill 18. 10.2807/ese.18.05.20394-en 23399422

[32] Cheng AC, Brown SG, Waterer GW, Holmes M, Senenayake S, et al (2013) Influenza epidemiology, vaccine coverage and vaccine effectiveness in sentinel Australian hospitals in 2012: the Influenza Complications Alert Network (FluCAN). Communicable Diseases Intelligence 37: E246–E252. 24890961 10.33321/cdi.2013.37.37

[33] Cheng AC, Holmes M, Irving LB, Brown SG, Waterer GW, et al (2013) Influenza Vaccine Effectiveness against Hospitalisation with Confirmed Influenza in the 2010–11 Seasons: A Test-negative Observational Study. PLoS One 8: e68760. 23874754 10.1371/journal.pone.0068760 PMC3712933

[34] Rothman KJ, Greenland S, Lash TL (2008) Case-Control studies. Modern epidemiology. Philadelphia: Wolters Kluwer Health/Lippincott Williams & Wilkins. 111–127.

[35] Sullivan SG, Tay EL, Kelly H (2013) Variable definitions of the influenza season and their impact on vaccine effectiveness estimates. Vaccine 31: 4280–4283. 23850417 10.1016/j.vaccine.2013.06.103

[36] Kirkwood BR, Sterne JAC (2003) Analysis of clustered data. Essential medical statistics. Malden, MA: Blackwell Science. 355–370.

[37] Puig-Barbera J, Natividad-Sancho A, Calabuig-Perez J, Lluch-Rodrigo JA, Pastor-Villalba E, et al (2013) MF59-adjuvanted and virosomal influenza vaccines for preventing influenza hospitalization in older people: comparative effectiveness using the Valencia health care information system. Vaccine 31: 3995–4002. 23731629 10.1016/j.vaccine.2013.05.070

[38] Smith DJ, Forrest S, Ackley DH, Perelson AS (1999) Variable efficacy of repeated annual influenza vaccination. Proc Natl Acad Sci U S A 96: 14001–14006. 10570188 10.1073/pnas.96.24.14001 PMC24180

[39] Anton A, Pozo F, Niubo J, Casas I, Pumarola T (2012) Influenza A(H1N1)pdm09 virus: viral characteristics and genetic evolution. Enferm Infecc Microbiol Clin 30 Suppl 4 10–17. 10.1016/S0213-005X(12)70099-X 23116787

[40] Daniels R, Gregory V, McCauley J (2013) Surveillance report. Influenza virus characterisation. Summary Europe, July 2013. Stockholm: European Centre for Disease Prevention and Control.

[41] Talbot HK, Zhu Y, Chen Q, Williams JV, Thompson MG, et al (2013) Effectiveness of influenza vaccine for preventing laboratory-confirmed influenza hospitalizations in adults, 2011–2012 influenza season. Clin Infect Dis 56: 1774–1777. 23449269 10.1093/cid/cit124 PMC10941295

[42] Treanor JJ, Talbot HK, Ohmit SE, Coleman LA, Thompson MG, et al (2012) Effectiveness of seasonal influenza vaccines in the United States during a season with circulation of all three vaccine strains. Clin Infect Dis 55: 951–959. 22843783 10.1093/cid/cis574 PMC3657521

[43] Beyer WE, McElhaney J, Smith DJ, Monto AS, Nguyen-Van-Tam JS, et al (2013) Cochrane re-arranged: support for policies to vaccinate elderly people against influenza. Vaccine 31: 6030–6033. 24095882 10.1016/j.vaccine.2013.09.063

