# Correction: 2012-2013 Seasonal Influenza Vaccine Effectiveness against Influenza Hospitalizations: Results from the Global Influenza Hospital Surveillance Network

**DOI:** 10.1371/journal.pone.0107849

**Published:** 2014-09-08

**Authors:** The PLOS ONE Staff

There is an error in Table 6. The heading in the 2^nd^ row, 6^th^ column should read “n” instead of “N.” Please see the corrected Table 6 here.

**Table 6:** Pooled IVE in hospitalized patients swabbed within 7 days of symptom onset.

		Influenza positive			Influenza negative			IVE adjusted for site a			Fully adjusted IVE a ^,^ b		
Influenza strain	Age group	n	N	%	n	N	%	IVE	95%CI		IVE	95%CI	
All influenza ^c^	Overall	132	675	20%	641	1509	42%	44%	27%	57%	33%	11%	49%
	<65 y	21	433	5%	95	632	15%	51%	15%	72%	35%	-15%	63%
	≥65 y	111	242	46%	546	877	62%	43%	22%	58%	31%	4%	51%
A(H1N1)	Overall	33	286	12%	641	1509	42%	52%	25%	70%	23%	-26%	53%
	<65 y	8	232	3%	95	632	15%	59%	7%	82%	40%	-43%	75%
	≥65 y	25	54	46%	546	877	62%	34%	-22%	64%	13%	-68%	55%
A(H3N2)	Overall	22	102	22%	641	1509	42%	33%	-23%	64%	30%	-37%	64%
	<65 y	5	63	8%	95	632	15%	18%	-147%	73%	19%	-162%	75%
	≥65 y	17	39	44%	546	877	62%	46%	-16%	75%	39%	-40%	73%
B/Yamagata	Overall	70	195	36%	641	1509	42%	43%	20%	59%	43%	17%	60%
	<65 y	6	60	10%	95	632	15%	58%	-5%	83%	52%	-25%	81%
	≥65 y	64	135	47%	546	877	62%	46%	23%	63%	41%	12%	61%

n, number of vaccinated patients in the group; N, total number of patients in the group; CI, confidence interval; IVE, influenza vaccine effectiveness.

a Site as a random effect.

b Adjusted by week of symptom onset, age group, sex, hospitalization in the previous 12 months, presence of chronic conditions, and smoking habits.
