# Supplementary figures and images for: 2012-2013 Seasonal Influenza Vaccine Effectiveness against Influenza Hospitalizations: Results from the Global Influenza Hospital Surveillance Network
Source: PLoS One. 2014 Jun 19;9(6):e100497. doi: 10.1371/journal.pone.0100497 (PMC4063939; doi:10.1371/journal.pone.0100497)

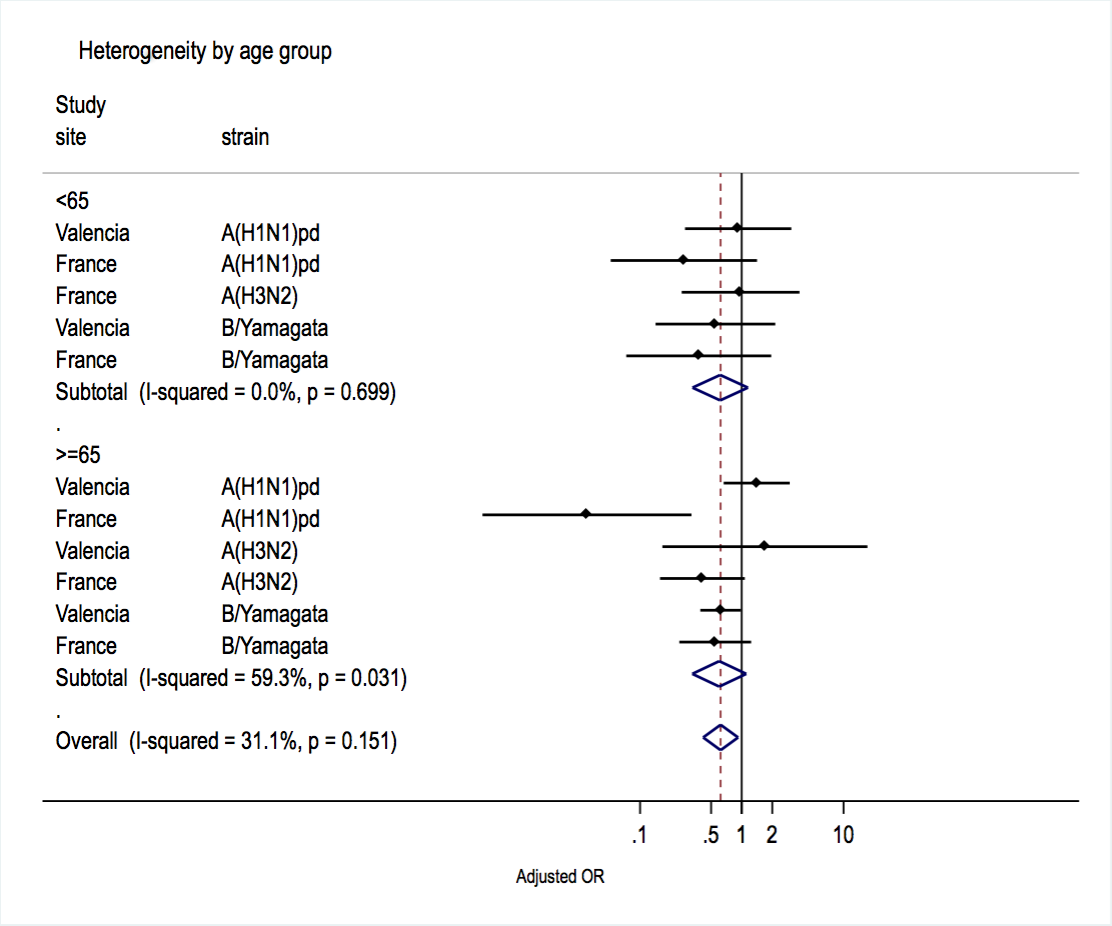

Supplement: Figure S1 — Heterogeneity in adjusted IVE estimates by age group. (TIF) [file pone.0100497.s001.tif]

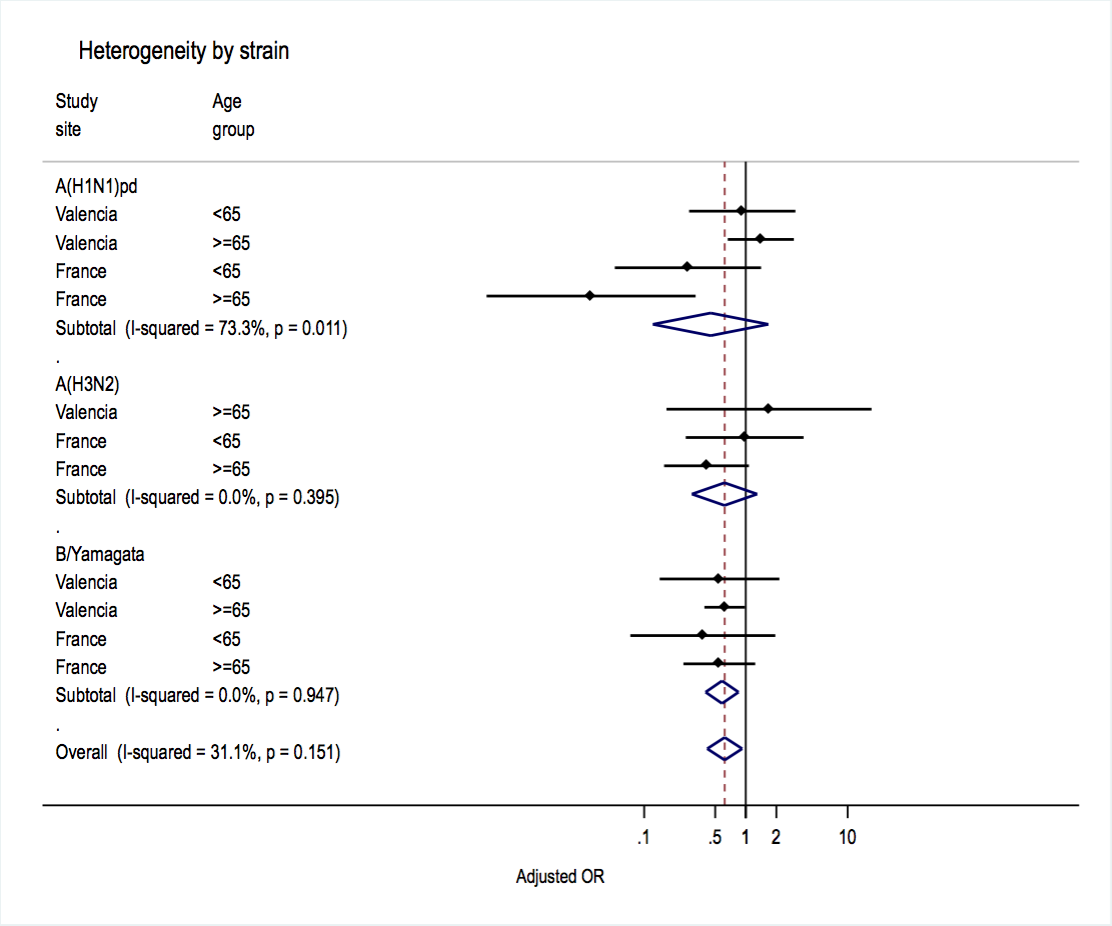

Supplement: Figure S2 — Heterogeneity in adjusted IVE estimates at each site by strain. (TIF) [file pone.0100497.s002.tif]

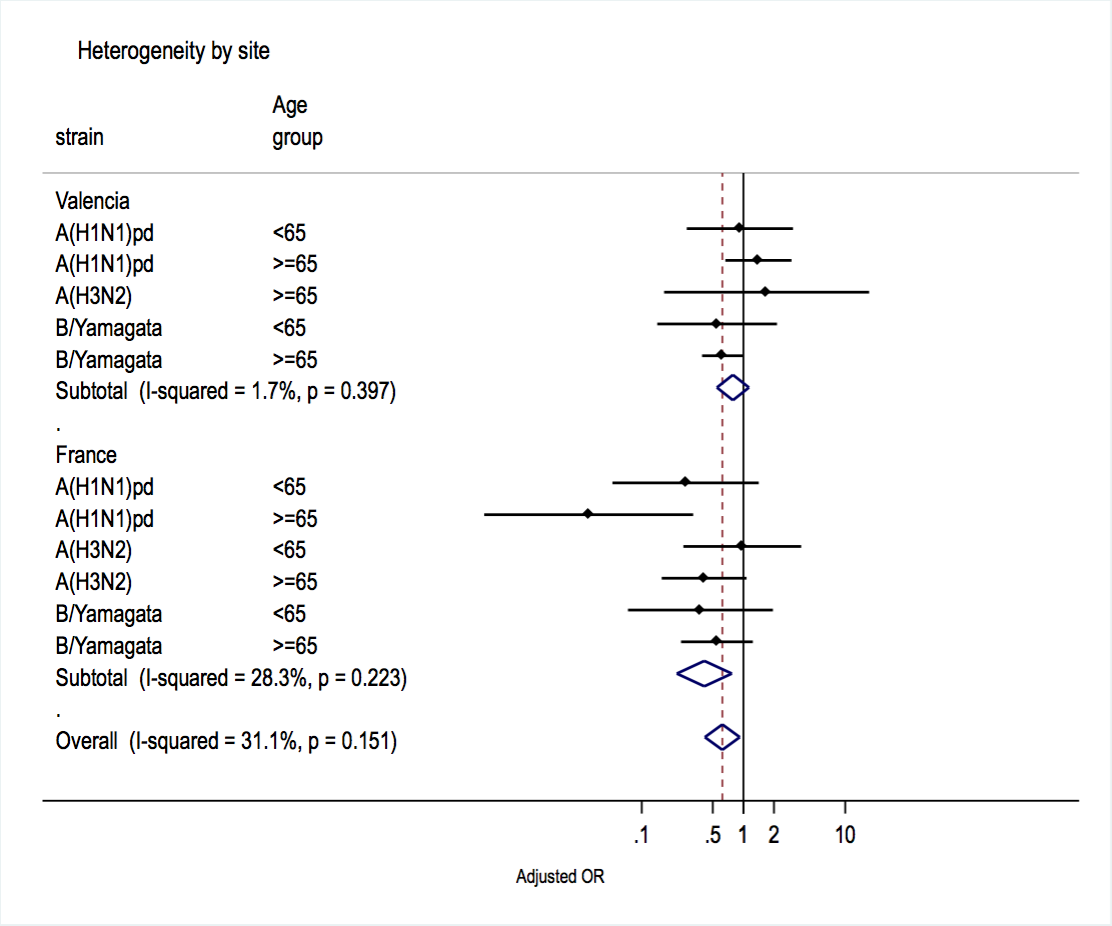

Supplement: Figure S3 — Heterogeneity in adjusted IVE estimates by site. (TIF) [file pone.0100497.s003.tif]
