# Supplementary material for: 2012-2013 Seasonal Influenza Vaccine Effectiveness against Influenza Hospitalizations: Results from the Global Influenza Hospital Surveillance Network
Source: PLoS One. 2014 Jun 19;9(6):e100497. doi: 10.1371/journal.pone.0100497 (PMC4063939; doi:10.1371/journal.pone.0100497)
Supplement: Table S1 — Hospital characteristics, inclusion and exclusion criteria, and influenza season definition for each site. (DOC) [file pone.0100497.s004.doc]

Table S1. Hospital characteristics, inclusion and exclusion criteria, and influenza season definition for each site

| **Study site** | **Hospital characteristics** | **Inclusion criteria^a^** | **Exclusion criteria** | **Study influenza season definition** |
| --- | --- | --- | --- | --- |
| Valencia | Five general hospitals serving a defined population (healthcare district) | Hospitalized during visits to emergency wards and for ≤ 48 h. Admission diagnosis associated with influenza infection | ILI symptoms >7 days; living outside catchment area; institutionalized; discharged from hospital in the previous 30 days; influenza vaccine contraindicated; previous confirmed influenza in the season. | Start: 2 consecutive weeks with ≥ 1 case per week End: 2 consecutive weeks with no cases |
| France | Five general university hospitals serving a population not specifically defined | Hospitalized for acute respiratory infection at participating wards ^b^ for ≥ 24 h | ILI symptoms >7 days; institutionalized; hospitalized for respiratory infection in the season; influenza vaccine contraindicated; previous confirmed influenza in the season; not affiliated with Social Security. | Influenza season defined by the national surveillance system |
| Moscow | One hospital for infectious disease, reference hospital for severe respiratory infections serving adults and children for the whole city | Hospitalized for acute respiratory infection for ≤ 48 h at participating wards | ILI symptoms >7 days; institutionalized; discharged from hospital in the previous 30 days | Start: week following the week in which 5 laboratory-confirmed cases of influenza were detected End: week during which no influenza cases were detected |
| St. Petersburg | Three reference hospitals for children and adults with severe respiratory infections serving the whole city | Hospitalized for acute respiratory infection for ≤ 48 h at participating wards | ILI symptoms >7 days; institutionalized; discharged from hospital in the previous 30 days | Start: week following the week in which 5 laboratory-confirmed cases of influenza were detected End: week during which no influenza cases were detected |

^a^ Possible diagnoses for inclusion are summarized in Table S5.

^b^ Participating wards were as follows: Hopital Cochin, emergency medicine, internal medicine, pneumology, oncology, and maternity; Hopital Bichat, emergency medicine, geriatrics, internal medicine, pneumology, dermatology, infectious diseases, intensive care, rheumatology, and liver-stomach-gastroenterology; Hopital Limoges: emergency medicine, geriatrics, internal medicine, cardiology, dermatology, pneumology, infectious diseases, nephrology, intensive care, and urology; Hopital Montpellier, emergency medicine, internal medicine, cardiology, dermatology, infectious diseases, nephrology, intensive care, urology, and maternity; Hopital Lyon, emergency medicine and geriatrics
