# Supplementary material for: 2012-2013 Seasonal Influenza Vaccine Effectiveness against Influenza Hospitalizations: Results from the Global Influenza Hospital Surveillance Network
Source: PLoS One. 2014 Jun 19;9(6):e100497. doi: 10.1371/journal.pone.0100497 (PMC4063939; doi:10.1371/journal.pone.0100497)
Supplement: Table S2 — Vaccination policies and vaccines available at each coordinating site. (DOC) [file pone.0100497.s005.doc]

**Table S2. Vaccination policies and vaccines available at each coordinating site**

| **Coordinating site(s)** | **Vaccines used** | **National Vaccination Policy** |
| --- | --- | --- |
| Valencia (Spain) | - **Unadjuvanted vaccine:** Vacuna Antigripal Pasteur | Offered free to health district inhabitants >6 months of age with high-risk conditions, ≥60 years of age with or without high-risk conditions, and all institutionalized individuals, pregnant women, and health workers |
| St. Petersburg & Moscow (Russian Federation) | - **Adjuvanted vaccines (produced in Russia):** Grippol® for adults and Grippol® Plus for children ≥6 years of age - **Unadjuvanted vaccines (not produced in Russia):** Influvac®, Agripal®, Inflexal®, Begrivac®, Vaxigrip®, Fluorix®, and Fluvaxin® | Highly recommended for children 0–6 months of age, schoolchildren, students, people in close contact with many people due to professional duties (medical workers, commerce, transport, etc.), living in crowded environments, community (reservists and etc.), with chronic diseases, or ≥60 years of age.  Vaccines produced in Russia are distributed free for at risk-groups. Imported vaccines are available for purchase. |
| France | - **Unadjuvanted vaccines:** Agrippal®, Fluarix®, Immugrip®, Inﬂuvac®, Vaxigrip®, Intanza® (18–59 years), Gripguard® (≥65 years), and Fluenz® (25 months–17 years) | Highly recommended for ≥65 years of age, pregnant women, patients ≥6 months of age with chronic disease, obese individuals, health workers, families with a child <6 months of age at risk for severe influenza infection |
