# Supplementary material for: 2012-2013 Seasonal Influenza Vaccine Effectiveness against Influenza Hospitalizations: Results from the Global Influenza Hospital Surveillance Network
Source: PLoS One. 2014 Jun 19;9(6):e100497. doi: 10.1371/journal.pone.0100497 (PMC4063939; doi:10.1371/journal.pone.0100497)
Supplement: Table S3 — Characteristics of patients included in the IVE analysis by site and influenza infection status. (DOC) [file pone.0100497.s006.doc]

**Table S3. Characteristics of patients included in the IVE analysis by site and influenza infection status**

| **Characteristic** | **Valencia** | | **St Petersburg** | | **Moscow** | | **France** | | **Overall** | |  |
| --- | --- | --- | --- | --- | --- | --- | --- | --- | --- | --- | --- |
|  | **Negative** | **Positive** | **Negative** | **Positive** | **Negative** | **Positive** | **Negative** | **Positive** | **Negative** | **Positive** |  |
|  | **N=852** | **N=170** | **N=39** | **N=82** | **N=369** | **N=301** | **N=249** | **N=122** | **N=1509** | **N=675** | **P-value** |
| Age group |  |  |  |  |  |  |  |  |  |  |  |
| 18-49 y | 5.4% | 11.8% | 30.8% | 30.5% | 85.1% | 87.7% | 14.1% | 23.8% | 27.0% | 50.1% | <0.0001 |
| 50-64 y | 14.4% | 10.0% | 41.0% | 48.8% | 9.8% | 6.3% | 20.1% | 15.6% | 14.9% | 14.1% |  |
| 65-74 y | 21.1% | 26.5% | 15.4% | 9.8% | 2.7% | 3.3% | 17.3% | 18.0% | 15.8% | 12.6% |  |
| 75-84 y | 35.7% | 32.4% | 10.3% | 9.8% | 1.9% | 2.3% | 25.7% | 25.4% | 25.1% | 15.0% |  |
| ≥ 85 y | 23.4% | 19.4% | 2.6% | 1.2% | 0.5% | 0.3% | 22.9% | 17.2% | 17.2% | 8.3% |  |
|  |  |  |  |  |  |  |  |  |  |  |  |
| Sex |  |  |  |  |  |  |  |  |  |  | <0.0001 |
| Female | 44.5% | 49.4% | 56.4% | 57.3% | 78.6% | 86.0% | 43.4% | 54.9% | 53.0% | 67.7% |  |
| Male | 55.2% | 50.6% | 43.6% | 42.7% | 21.4% | 14.0% | 56.6% | 45.1% | 47.0% | 32.3% |  |
|  |  |  |  |  |  |  |  |  |  |  |  |
| Comorbidities |  |  |  |  |  |  |  |  |  |  |  |
| 0 | 11.3% | 22.9% | 12.8% | 26.8% | 66.9% | 72.4% | 11.2% | 14.8% | 24.9% | 44.0% | <0.0001 |
| 1 | 36.9% | 37.7% | 64.1% | 51.2% | 24.9% | 22.3% | 32.5% | 37.7% | 33.9% | 32.4% |  |
| ≥2 | 51.9% | 39.4% | 23.1% | 22.0% | 8.1% | 5.3% | 56.2% | 47.5% | 41.2% | 23.6% |  |
|  |  |  |  |  |  |  |  |  |  |  |  |
| Obese (body mass index ≥30) | 32.6% | 34.7% | 30.8% | 45.1% | 22.5% | 14.3% | 21.7% | 19.7% | 28.3% | 24.2% | 0.1150 |
|  |  |  |  |  |  |  |  |  |  |  |  |
| Hospitalized in the last 12 months ^a^ | 37.9% | 27.7% | 13.3% | 11.4% | 9.5% | 6.3% | 44.6% | 41.8% | 32.0% | 18.8% | <0.0001 |
|  |  |  |  |  |  |  |  |  |  |  |  |
| General practitioner visits in the last 3 months ^b^ |  |  |  |  |  |  |  |  |  |  |  |
| 0 | 25.0% | 30.0% | 64.5% | 69.6% | 90.2% | 89.7% | 0.5% | 0.0% | 38.8% | 58.0% | <0.0001 |
| 1 | 26.4% | 24.1% | 22.6% | 20.3% | 4.3% | 7.0% | 33.7% | 39.8% | 21.8% | 18.1% |  |
| ≥2 | 48.6% | 45.9% | 12.9% | 10.1% | 5.4% | 3.3% | 65.9% | 60.2% | 39.4% | 23.9% |  |
|  |  |  |  |  |  |  |  |  |  |  |  |
| Smoking |  |  |  |  |  |  |  |  |  |  |  |
| Never | 44.0% | 51.2% | 74.4% | 69.5% | 56.4% | 60.8% | 45.4% | 47.5% | 48.1% | 57.0% | <0.0001 |
| Past | 40.0% | 28.2% | 0.0% | 4.9% | 27.1% | 27.6% | 33.7% | 27.1% | 34.8% | 24.9% |  |
| Current | 16.0% | 22.6% | 25.6% | 25.6% | 16.5% | 11.6% | 20.9% | 25.4% | 17.2% | 18.1% |  |
|  |  |  |  |  |  |  |  |  |  |  |  |
| Socioeconomic class |  |  |  |  |  |  |  |  |  |  |  |
| Professional to non-manual-skilled | 13.2% | 12.4% | 38.5% | 19.5% | 67.2% | 64.8% | 10.4% | 19.7% | 26.6% | 37.9% | <0.0001 |
| Manual-skilled | 14.9% | 17.7% | 10.3% | 11.0% | 12.2% | 8.6% | 0.8% | 1.6% | 11.8% | 9.9% |  |
| Manual-non-skilled | 71.4% | 70.0% | 12.8% | 12.2% | 6.8% | 4.7% | - | - | 42.3% | 21.2% |  |
| Unknown | 0.6% | 0.0% | 38.5% | 57.3% | 13.8% | 21.9% | 88.8% | 78.7% | 19.4% | 31.0% |  |
|  |  |  |  |  |  |  |  |  |  |  |  |
| Functional capacity |  |  |  |  |  |  |  |  |  |  |  |
| No impairment ^c^ | 62.5% | 70.7% | 40.0% | 40.0% | - | - | 65.2% | 64.9% | 62.9% | 67.3% | 0.2320 |
|  |  |  |  |  |  |  |  |  |  |  |  |
| Influenza vaccine |  |  |  |  |  |  |  |  |  |  |  |
| 2012/2013 | 57.3% | 45.9% | 5.1% | 2.4% | 0.5% | 1.0% | 59.8% | 40.2% | 42.5% | 19.6% | <0.0001 |
| 2011/2012 ^d^ | 54.0% | 44.1% | 2.6% | 1.2% | 5.4% | 5.0% | 63.1% | 50.0% | 42.3% | 22.5% | <0.0001 |
|  |  |  |  |  |  |  |  |  |  |  |  |
| Onset to swabbing |  |  |  |  |  |  |  |  |  |  |  |
| 1 to 2 d | 22.7% | 21.8% | 20.5% | 34.2% | 46.9% | 54.5% | 29.7% | 25.4% | 29.7% | 38.5% | <0.0001 |
| 3 to 4 d | 46.7% | 50.0% | 48.7% | 45.1% | 34.2% | 35.6% | 39.4% | 44.3% | 42.5% | 41.9% |  |
| 5 to 7 d | 30.6% | 28.2% | 30.8% | 20.7% | 19.0% | 10.0% | 30.9% | 30.3% | 27.8% | 19.6% |  |

^a^ N=2171; 13 missing values

^b^ N=2108; 76 missing values

^c^ Barthel score > 60; N= 1119; 50 missing values

^d^ N= 2176; 8 missing values
