# Supplementary material for: 2012-2013 Seasonal Influenza Vaccine Effectiveness against Influenza Hospitalizations: Results from the Global Influenza Hospital Surveillance Network
Source: PLoS One. 2014 Jun 19;9(6):e100497. doi: 10.1371/journal.pone.0100497 (PMC4063939; doi:10.1371/journal.pone.0100497)
Supplement: Table S4 — Pooled IVE in hospitalized patients swabbed within 4 days of symptom onset. (DOC) [file pone.0100497.s007.doc]

**Table S4**. Pooled IVE in hospitalized patients swabbed within 4 days of symptom onset

| **Influenza strain/age group** | **Influenza positive** | | |  | **Influenza negative** | | |  | **IVE adjusted for site ^a^** | | |  | **Fully adjusted IVE ^a,b^** | | |
| --- | --- | --- | --- | --- | --- | --- | --- | --- | --- | --- | --- | --- | --- | --- | --- |
|  | **n** | **N** | **%** |  | **n** | **N** | **%** |  | **IVE** | **95%CI** |  |  | **IVE** | **95%CI** |  |
| All influenza ^c^ |  |  |  |  |  |  |  |  |  |  |  |  |  |  |  |
| Overall | 97 | 543 | 18% |  | 460 | 1089 | 42% |  | 47% | 27% | 61% |  | 34% | 8% | 53% |
| <65 y | 16 | 367 | 4% |  | 68 | 470 | 14% |  | 56% | 16% | 77% |  | 40% | -17% | 69% |
| ≥65 y | 81 | 176 | 46% |  | 392 | 619 | 63% |  | 41% | 15% | 60% |  | 29% | -6% | 52% |
| A(H1N1) |  |  |  |  |  |  |  |  |  |  |  |  |  |  |  |
| Overall | 26 | 251 | 10% |  | 460 | 1089 | 42% |  | 56% | 26% | 74% |  | 24% | -35% | 57% |
| <65 y | 6 | 210 | 3% |  | 68 | 470 | 14% |  | 69% | 22% | 88% |  | 55% | -24% | 84% |
| ≥65 y | 20 | 41 | 49% |  | 392 | 619 | 63% |  | 14% | -82% | 59% |  | -14% | -158% | 49% |
| A(H3N2) |  |  |  |  |  |  |  |  |  |  |  |  |  |  |  |
| Overall | 15 | 80 | 19% |  | 460 | 1089 | 42% |  | 34% | -42% | 69% |  | 34% | -53% | 71% |
| <65 y | 5 | 53 | 9% |  | 68 | 470 | 14% |  | -25% | -322% | 63% |  | -31% | -405% | 66% |
| ≥65 y | 10 | 27 | 37% |  | 392 | 619 | 63% |  | 58% | -11% | 84% |  | 56% | -34% | 84% |
| B/Yamagata |  |  |  |  |  |  |  |  |  |  |  |  |  |  |  |
| Overall | 50 | 139 | 36% |  | 460 | 1089 | 42% |  | 46% | 20% | 64% |  | 45% | 15% | 65% |
| <65 y | 4 | 42 | 10% |  | 68 | 470 | 14% |  | 65% | -9% | 89% |  | 63% | -19% | 88% |
| >=65 | 46 | 97 | 47% |  | 392 | 619 | 63% |  | 48% | 20% | 67% |  | 41% | 3% | 63% |

^a^ Site as a random effect

^b^ Adjusted by week of symptom onset, age group, sex, hospitalization in the previous 12 months, presence of chronic conditions, and smoking habits

^c^ Included all identified strains: A(H1N1)pdm09 (n=283), A(H3N2) (n=102), A untyped (n=25), B/Yamagata (n=195), B/Victoria (n=18), B untyped (n=52).
