# Supplementary material for: 2012-2013 Seasonal Influenza Vaccine Effectiveness against Influenza Hospitalizations: Results from the Global Influenza Hospital Surveillance Network
Source: PLoS One. 2014 Jun 19;9(6):e100497. doi: 10.1371/journal.pone.0100497 (PMC4063939; doi:10.1371/journal.pone.0100497)
Supplement: Table S5 — Diagnosis codes used to identify emergency admissions possibly associated with an influenza infection and considered for inclusion. (DOC) [file pone.0100497.s008.doc]

**Table S5. Diagnosis codes used to identify admissions possibly associated with an influenza infection and considered for inclusion**

| **Criteria** | **Valencia** | **St. Petersburg** | **Moscow** | **France** | **ICD 9 Codes** | **ICD 10 Codes** |
| --- | --- | --- | --- | --- | --- | --- |
| Acute respiratory infection | X | X | X | X | 382.9; 460-466 | J00-J06, J20-J22, H66.90 |
| Pneumonia and influenza | X | X | X | X | 480-488 | J09-J18 |
| Acute myocardial infarction or acute coronary syndrome | X |  |  | X | 410-411 and 413-414 | I20-I25.9 |
| Asthma | X |  |  | X | 493-493.92 | J45.2-J45.22, J45.9-J45.998, J44-J44.9 |
| Heart failure | X |  |  | X | 428-429.0 | I50-I50.9; I51.4 |
| Chronic obstructive pulmonary disease | X |  |  | X | 490, 491, 492, 496 | J40-J44.9 |
| Myalgia | X |  |  | X | 729.1 | M79.1 |
| Metabolic failure (diabetic coma, renal dysfunction, acid-base disturbances, alterations to the water balance) | X |  |  | X | 250.1- 250.3; 584-586; 276-277 | E11.9, E10.9, E11.65, E10.65, E10.11, E11.01, E10.641, E11.641, E10.69, E11.00, E10.10, E11.69, N17.0, N17.1, N17.2, N17.8, N17.9, N18.1, N18.2, N18.3, N18.4, N18.5, N18.6M N18.9, N19, E87.0, E87.1, E87.2, E87.3, E87.4, E87.5, E87.6, E87.70, E87.71, E87.79, E86.0, E86.1 |
| Altered consciousness, convulsions, febrile-convulsions | X |  |  | X | 780.01-780.02; 780.09; 780.31-780.32 | R40.20, R40.4, R40.0, R40.1, R56.00, R56.01 |
| Dyspnea/respiratory abnormality | X |  |  | X | 786.0 | R06.0, R06-R06.9 |
| Respiratory abnormality | X |  |  | X | 786.00 | R06.9 |
| Shortness of breath | X |  |  | X | 786.05 | R06.02 |
| Respiratory abnormality nec | X |  |  | X | 786.09 | R06.3, R06.00, R06.09, R06.83 |
| Respiratory symptoms/chest symptoms | X |  |  | X | 786.9 | R06.89 |
| Fever or fever unknown origin or non-specified | X |  |  | X | 780.6-780.60 | R50, R50.9 |
| Cough | X |  |  | X | 786.2 | R05 |
| Sepsis, Systemic inflammatory response syndrome | X |  |  | X | 995.90-995.94 | R65.10, R65.11, R65.20, A41.9 |

X indicates that the diagnosis code was used at the indicated coordinating site.
